# First reported stevia-containing human urinary calculi

**DOI:** 10.1016/j.eucr.2026.103563

**Published:** 2026-07-28

**Authors:** Sara L. Best, John R. Asplin, Natalia Gromadzka, Benjamin C. Owen, Yongbo Zhang, Valentinas Gruzdys

**Affiliations:** a Department of Urology, University of Wisconsin, School of Medicine and Public Health, Madison, WI, USA; b Litholink, Laboratory Corporation of America, Itasca, IL, USA; c Department of Chemistry, Integrated Molecular Structure Education and Research Center, Northwestern University, Evanston, IL, USA

**Keywords:** Nephrolithiasis, Stevia, Supplements, Urinary calculi

## Abstract

While most kidney stones are made of typical substances like calcium oxalate, patients with uncommon metabolic disorders or exposures/ingestions can crystallize rare materials in their urinary tracts, leading to unusual stone compositions. Six stones in 5 individuals were evaluated when their composition did not match known lithogenic substances. We report the first ever identified human urinary calculi composed of steviol, a metabolite of the no-calorie sweetener, stevia.

## 1 Introduction

While most human urinary stones are composed of typical substances such as calcium oxalate, uric acid, and struvite, the identification of rare substances in stones is often critical for understanding the mechanism of stone formation and the subsequent development of a treatment plan. This is particularly true when stones contain crystals stemming from exposure to exogenous substances such as medications or supplements, substances consumed with the intention of a “health benefit.” For example, a patient who forms a stone containing triamterene may be better served by being switched to an alternative diuretic medication.

In late 2024, our stone analysis lab received several urinary calculi that contained a previously-unidentified substance that ultimately proved to be steviol, the molecular backbone of the no-calorie sweetener stevia. We present the methods used to confirm the composition of the steviol urinary stones, the infrared (IR) spectra of the stones and the dietary exposures associated with them.

## 2 Case presentations

Between June 2024 and June 2025, our laboratory (Litholink, a division of Labcorp, Itasca, IL) received 6 urinary stones from 5 different patients containing a substance that did not match any other known substance in our IR spectral library. Briefly, stones were processed for analysis by pulverization to a fine powder and then analyzed by IR spectroscopy and the resultant spectrum compared to our proprietary spectral library of known substances. Four specimens contained typical human urinary stone components such as calcium oxalate or calcium phosphate in addition to the unknown substance, while two stones were composed solely of the unknown material.

**Fig. 1:**
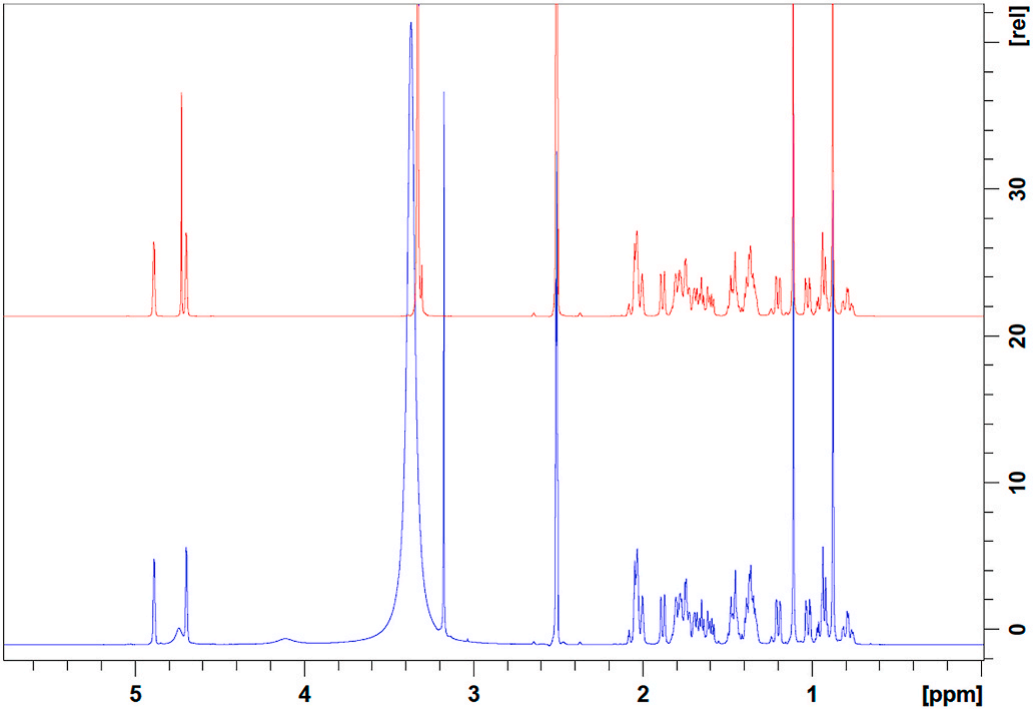
Proton (^1^H) NMR spectra comparing steviol standard (top, red) to steviol stone material (bottom, blue). Y-axis – relative peak intensity; X-axis – chemical shift (ppm).

**Fig. 2:**
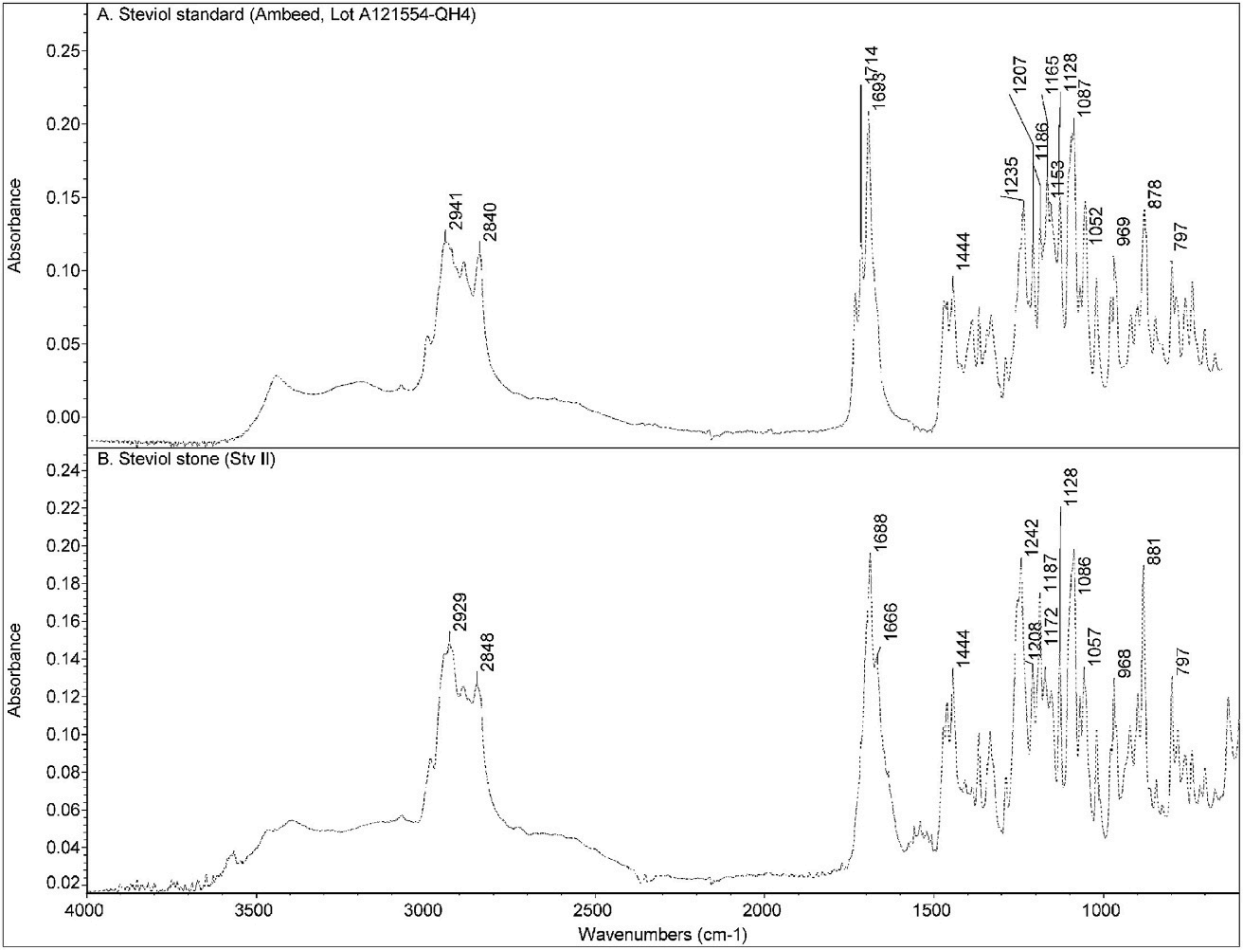
Infrared spectra of A) steviol standard B) pure steviol stone (Stv II).

Using methodology we have previously described,^1^ stone material from two patients, both composed of a mix of calcium oxalate and unknown material were submitted for identification using liquid chromatography-high-resolution mass spectrometry (LC-MS), tandem MS, and nuclear magnetic resonance (NMR). The MS data showed a protonated ion at a *m*/*z* (mass-to-charge ratio) of 319.2273, matching the empirical formula C_20_H_30_O_3_ (1.6 ppm) for hydroxydehydrostevic acid, better known as steviol (318.4 g/mol molecular weight without protonation). Unknown material proton NMR also matched steviol standard chemical shifts (Fig. 1). From this work, we were able to identify the unknown substance to be steviol, the backbone of the sweetener stevia. In addition, a steviol reference standard was obtained and analyzed with infrared spectroscopy, with the resulting spectrum matching that of the spectra seen in our original unknown stone samples (Fig. 2).

Patients with stones containing steviol were then contacted and a limited dietary history was obtained from 4 out of 5 patients, focusing on their consumption of products containing stevia. Interviews revealed that all affected patients were purchasing stevia sweeteners and using them to sweeten their foods and beverages (Table 1). Two patients reported buying powdered stevia in bulk while the other 2 purchased liquid formulations. One patient also consumed other stevia-containing products such as stevia-sweetened yogurt or soda once or twice a day, in addition to sweetening other drinks with liquid stevia. Patients' main stevia intakes appeared to come from the bulk powder or liquid formulations. By determining which product they were consuming and how often they were re-purchasing it, we were able to estimate how many of the manufacturer-reported stevia servings each patient was consuming per day. For example, if a patient consumes 3 bottles of liquid stevia reported to contain 80 servings each in a week (240 servings), we estimated the patient's daily consumption at 34 servings/day.

**Table 1:** Steviol-containing stones and associated patient information.

	Gender	Date	Age	Source	Stone Weight	Stone Composition	Stevia Exposure
Stv I	F	10/2024	60	Kidney	1482mg	30% COM, 10%CaPhos, 60% Steviol	34 servings/day
Stv II	F	11/2024	61	Kidney	189mg	100% Steviol	>88 servings/day
Stv III	F	11/2024	46	N/A	148mg	30% CaPhos, 70% Steviol	60 servings/day
Stv IV^a^	M	6/2024	71	Bladder	412mg	100% Steviol	68 servings/day
Stv V	F	2/2025	76	N/A	65mg	60% COM, 20% COD, 20% Steviol	N/A
Stv VI^a^	M	6/2025	72	Bladder	33,344 mg	20% CaPhos, 80% Steviol	68 servings/day

a Stones were received from the same patient(COM = Calcium Oxalate Monohydrate, CaPhos = Calcium Phosphate).

This study was conducted under the institutional review board oversight (SCMM-RND-402 WCG IRB Protocol #520180046). The work involved analysis of kidney stone specimens with limited associated, nonidentifiable information; therefore, the study met criteria for exemption under the US Federal Policy for the Protection of Human Subjects (45 CFR 46.104(d)). Patients contacted for dietary history provided verbal consent before providing this information.

## 3 Discussion

The last 50 years have provided modern consumers a wide selection of non-caloric sweeteners to choose from, including saccharin, aspartame, and sucralose. One of these zero-calorie sweeteners, stevia, was first recognized by the FDA as a dietary supplement in 1995, but it has been harvested from its main source plant, *Stevia rebaudiana*, by South Americans for centuries.^2^

*Stevia rebaudiana* is a perennial plant native to parts of South America, including Brazil and Paraguay. While it has been used for its sweet taste for centuries by people native to the area, the compounds responsible for this sweetness, mainly stevioside and rebaudioside, were first identified by French chemists in 1931.^3^^,^^4^ While the terminology and American regulatory history for stevia are complex, the Food and Drug Administration (FDA) began permitting stevia to be used as a dietary supplement in 1995.^2^ In 2008, the FDA offered “no objection” to Generally Recognized as Safe (GRAS) status for rebaudioside A products Truvia® and PureVia®, classifying them not as “stevia” but as high-purity extracts, and as of 2017, certain high-purity stevia-related extracts may be sold as food additives in the US.^2^

The determination of “safe dosing” for a sweetener like steviol is interesting in and of itself. The FDA references a methodology proposed by Renwick,^5^ where the author gathered previously published data on consumption of other no-calorie sweeteners such as aspartame and saccharin in “average consumers” and “high consumers” in mg/kg body weight. He then converted these to “sucrose equivalents” by multiplying the amount of non-caloric sweetener consumed by a “sweetness potency” to predict how much sugar the alternative sweetener is replacing in a person's diet. For example, aspartame is said to be 180 times sweeter than sucrose while saccharin is 300 times sweeter. By combining the overall “sucrose equivalents” consumed in his meta-analysis of alternative sweetener consumption papers, Renwick estimated that the average non-diabetic consumer of no-calorie sweeteners consumed about 255mg/kg sucrose-equivalents per day, while a high consumer ate 675mg/kg/day. By using a “sweetness potency” ratio of 200 and the molecular weights of steviol and rebaudioside A, he estimated that the high consumer of stevia would be ingest 3.4mg/kg per day of rebaudioside A and 1.1mg/kg of steviol. While the FDA established “accepted daily intake (ADI)” levels for 6 other high intensity sweeteners such as aspartame, they instead reference the Joint Food and Agricultural Organization/World Health Organization Expert Committee on Food Additives (JECFA) ADI for steviol of 0-4mg/kg body weight.^6^^,^^7^

It is difficult, however, for a consumer to determine the dose of steviol they are consuming based on product labels. A liquid formulation one patient reported using states the serving size is “4 drops,” for example, but does not state how many milligrams of steviol these 4 drops contain, nor how this serving size was determined or whether it is based on any regulatory agency's “acceptable daily intake.” While it was impossible to calculate the exact steviol dose consumed daily by patients who formed a steviol urinary stone, all those we interviewed consume many (30-80) servings per day of their chosen stevia product, likely far more than the typical consumers.^5^ Whether the propensity for steviol stone formation is strictly dose-related is unknown, but it would seem plausible that the appearance of this poorly-soluble, non-glucuronidated stevia metabolite in the urine could be dose dependent.

Adding to the uncertainty of consumption is likely interpersonal variation in absorption. The main components of stevia, stevioside and rebaudioside A, are indigestible by human-produced enzymes alone, but are instead metabolized to steviol in the colon by gut bacteria.^8^ A study by Geuns et al. administered 250mg capsules of stevioside orally to healthy young volunteers thrice daily for 3 days and measured serum, stool and urine levels.^9^ No steviosides were detected in the fecal samples, only free steviol, suggesting complete degradation by colonic bacteria. They found no free steviol in either the urine or blood of the volunteers, instead finding only the glucuronidated form of steviol. The amounts of glucuronidated steviol detected in urine or blood at different time points varied dramatically between participants, suggesting significant variation in metabolism. This differs from the free steviol form found in the urinary stones we report, which makes some sense as it would be expected that the glucuronidated form of steviol would be highly soluble and unlikely to crystallize into calculi. Whether Geuns et al. did not detect free steviol in the urine because it was not there, or because its presence is dose dependent, or methodological factors such as steviol precipitation out of solution during collection preventing its measurement is unknown.

## 4 Conclusion

Steviol, a metabolite of the sweeteners in stevia, can crystallize and become a part of urinary stones. Whether this phenomenon is truly dose-dependent is unknown but interviewed patients all reported consuming dozens of servings per day of their stevia product of choice. We encourage other stone composition labs to add steviol to their IR spectral libraries so that the true incidence of this stone composition can be better elucidated and so that clinicians can counsel patients on dietary changes that may reduce their stone risk.

## Funding statement

This work made use of the IMSERC (RRID:SCR_017874) NMR and Mass Spectrometry (MS) facilities at Northwestern University, which have received support from the Soft and Hybrid Nanotechnology Experimental (SHyNE) Resource (NSF ECCS-2025633), NIH 1S10OD012016-01/1S10RR019071-01A1, and Northwestern University.

## CRediT authorship contribution statement

**Sara L. Best:** Conceptualization, Visualization, Writing – original draft, Writing – review & editing. **John R. Asplin:** Conceptualization, Supervision, Writing – review & editing. **Natalia Gromadzka:** Investigation, Writing – review & editing. **Benjamin C. Owen:** Investigation, Writing – review & editing. **Yongbo Zhang:** Investigation, Writing – review & editing. **Valentinas Gruzdys:** Investigation, Project administration, Writing – review & editing.
